# From commensalism to parasitism in Carapidae (Ophidiiformes): heterochronic modes of development?

**DOI:** 10.7717/peerj.1786

**Published:** 2016-03-10

**Authors:** Eric Parmentier, Déborah Lanterbecq, Igor Eeckhaut

**Affiliations:** 1 Laboratory of Functional & Evolutionary Morphology, AFFISH-RC, University of Liège, Liège, Belgium; 2 Biology of Marine Organisms and Biomimetics, University of Mons, Mons, Belgium; 3 Laboratoire de Biotechnologie et Biologie Appliquée, Haute Ecole Provinciale de Hainaut-Condorcet (& CARAH asbl), Ath, Belgium

**Keywords:** Molecular phylogeny, Character evolution, Symbiosis, Pearlfish, Echinoderms, *Carapus*, *Encheliophis*, Sound production, Ontogeny

## Abstract

Phenotypic variations allow a lineage to move into new regions of the adaptive landscape. The purpose of this study is to analyse the life history of the pearlfishes (Carapinae) in a phylogenetic framework and particularly to highlight the evolution of parasite and commensal ways of life. Furthermore, we investigate the skull anatomy of parasites and commensals and discuss the developmental process that would explain the passage from one form to the other. The genus *Carapus* forms a paraphyletic grouping in contrast to the genus *Encheliophis*, which forms a monophyletic cluster. The combination of phylogenetic, morphologic and ontogenetic data clearly indicates that parasitic species derive from commensal species and do not constitute an iterative evolution from free-living forms. Although the head morphology of *Carapus* species differs completely from *Encheliophis*, *C. homei* is the sister group of the parasites. Interestingly, morphological characteristics allowing the establishment of the relation between *Carapus homei* and *Encheliophis* spp. concern the sound-producing mechanism, which can explain the diversification of the taxon but not the acquisition of the parasite morphotype. *Carapus homei* already has the sound-producing mechanism typically found in the parasite form but still has a commensal way of life and the corresponding head structure. Moreover, comparisons between the larval and adult Carapini highlight that the adult morphotype “*Encheliophis*” is obtained by going beyond the adult stage reached by *Carapus*. The entrance into the new adaptive landscape could have been realised by at least two processes: paedomorphosis and allometric repatterning.

## Introduction

Adaptive radiation of a taxon results from morphological, physiological or behavioural modifications (Schluter, 2001), and the life histories of taxa are characterised by periodic introductions of novelties that have had significant effects on subsequent ecological and evolutionary diversity. The entrance of a phyletic lineage into a new adaptive landscape is linked to morphological modifications leading to a new kind of morphotype (Mayr, 1989). Minor modifications allow the specialisation of species into different ecological niches, promoting speciation. These modifications can result from one or several changes of an ancestral body plan (Zelditch & Fink, 1996) or from the emergence of novelties. Two main categories of innovations can be recognized in terms of how they impact the evolutionary dynamics and diversity of functional systems: those that directly influence the potential for phenotypic variation and those that allow the lineage to move into new regions of the adaptive landscape where new variants are favoured (Wainwright, 2007). Novelties can have various origins. Small changes in genes controlling ontogeny can induce large phenotypic variation and developmental heterochronies are thought to play an important role in both micro- and macro-evolutionary processes (Alberch et al., 1979; Gould, 1977). Heterochrony corresponds to changes in developmental rate or the relative time of appearance of features that link stages of ontogeny and phylogeny, for a given age, the descendant has a shape typical of the ancestor at a more juvenile age (paedomorphosis) or at a more mature age (peramorphosis) or retains ancestral shape but differs in size (Webster & Zelditch, 2005). However, some evolutionary modifications to ontogeny can lie beyond the realm of changes in developmental rate or timing and various non-heterochronic modes of developmental reprogramming have been proposed (Webster & Zelditch, 2005).

Among the diverse symbioses existing in the marine environment, one remarkable association is the one involving pearlfish of the Carapidae family as symbionts and various marine invertebrates as hosts (holothuroids, asteroids and bivalves). Carapidae Jordan & Fowler, 1902 belong to the Ophidiiformes and include two recognized subfamilies: the Pyramodontinae (two genera: *Snyderidia* Gilbert, 1905 and *Pyramodon* Smith & Radcliffe, 1913) and the Carapinae (Nielsen et al., 1999). Tetragondacninae (comprised of the monotypic genus *Tetragondacnus*) could constitute a third subfamily (Anderson & Satria, 2007), but additional data on the anatomy are required to assess its phylogenetic placement. Carapinae is divided into two tribes: the Echiodontini (three genera: *Eurypleuron* Markle & Olney, 1990, *Echiodon* Thompson, 1837 and *Onuxodon* Smith, 1955) and the Carapini (two genera: *Carapus* Rafinesque, 1810 and *Encheliophis* Muller, 1842). *Onuxodon*, *Carapus* and *Encheliophis* are well known for their unusual and notable behaviour, as they are able, depending on the species, to enter and reside in invertebrate hosts (Glynn et al., 2008; Parmentier, Castro-Aguirre & Vandewalle, 2000; Parmentier & Vandewalle, 2005; Trott, 1970; Trott, 1981). Symbiosis is unknown in other pearlfish genera. Species belonging to the genera *Onuxodon* and *Carapus* are commensals whereas *Encheliophis* species are regarded as parasites (Parmentier, Castro-Aguirre & Vandewalle, 2000; Parmentier & Das, 2004). Parasites reside most of the time inside their hosts and eat their internal tissues (gonads, digestive glands), while commensals use their hosts as shelters and feed outside the hosts (Parmentier & Das, 2004; Parmentier & Vandewalle, 2003). The difference in lifestyle behaviour is reflected in the buccal and pharyngeal jaw morphology. Commensals have extremely strong buccal elements, strong dentition, a wide mouth opening with jaw protrusion and a robust food intake apparatus. Parasites have a generally weak buccal apparatus and a narrow mouth opening, reflecting the less pronounced dietary constraints of their lifestyle: the jaws are more slender, and the insertions of the adductor mandibulae A_1_ along the entire length of the maxilla associated with the lack of mobility between the maxilla, preventing buccal protrusion (Parmentier et al., 1998; Parmentier & Vandewalle, 2003; Vandewalle et al., 1998).

Sound-producing muscles attached to swim bladder were found in all examined pearlfish (Parmentier, Chardon & Vandewalle, 2002; Parmentier & Diogo, 2006). One of the most notable characteristics of pearlfishes is the sounds they produce (Lagardere, Millot & Parmentier, 2005; Parmentier et al., 2006a; Parmentier et al., 2006b; Parmentier et al., 2008; Parmentier, Vandewalle & Lagardére, 2003). The recorded sounds appear species specific, indicating intraspecific selection for the sound-producing mechanisms and that the different species should be able to discriminate the calls. However, more information is needed on the behavioural patterns associated with sound production in carapid fishes.

The life cycle of Carapidae is divided into four stages: the vexillifer and tenuis larvae, juveniles and adults. The vexillifer larva corresponds to the dispersal pelagic stage (Olney & Markle, 1979). The tenuis larva is first marked by the loss of the vexillum and by a substantial lengthening. During their first contact with an invertebrate host, the tenuis larvae of *Carapus* and *Encheliophis* undergo an important shortening of the body, leading to the juvenile stage (Arnold, 1956; Padoa, 1947; Parmentier, Lecchini & Vandewalle, 2004), which gives it an adult-like morphology. Adults are similar in morphology to juveniles except that they are sexually mature.

The phylogenetic position of Carapidae within Ophidiiformes was confirmed based on the analysis of protein-coding mitochondrial DNA sequences (Miya et al., 2003) and the nuclear marker RNF213 (Li et al., 2009), but no molecular analyses have been conducted to infer the phylogenetic relationships within the group. The most recent phylogenetic hypothesis is based on an analysis of 38 morphological and behavioural characteristics (Parmentier et al., 2000a). Considering two carapid species, *Snyderidia canina* Gilbert, 1905 and *Onuxodon fowleri* (Smith, 1955) as the out-group, the inferred cladistic tree of the Carapini tribe showed the monophyly of the two recognized genera: *Carapus*, whose members are regarded as commensals, and *Encheliophis*, regarded as endoparasites (Parmentier & Das, 2004). *Carapus* species are distinguished by the following synapomorphies: (i) the presence of cardiform teeth with two to three enlarged teeth anteriorly on the premaxilla, and at least two rows of small conical teeth over the entire length of the premaxilla; (ii) the presence of an external row of conical teeth and several rows of internal smaller conical teeth on the dentary (Parmentier et al., 2000a). *Encheliophis* species later received more attention (Parmentier, 2004; Parmentier et al., 2010) leading to some modifications in the phylogenetic character matrix. This allowed improvement of the diagnosis, but did not change the phyletic relationships with *Carapus*. *Encheliophis* possess a single row of small, evenly spaced teeth on the dentary, maxilla and premaxilla bound by skin to the head, cardiform teeth on the premaxilla and, in certain cases, small conical teeth on the premaxilla. These fishes lack a maxillo-mandibular ligament (Parmentier et al., 2010). These results are in agreement with Trott’s hypothesis: *Encheliophis* may have evolved from a *Carapus*-like ancestor, using the host first as shelter and later as a food supply, leading to a change in the buccal structures in response to the new lifestyle (Trott, 1970). Carapini possess therefore, useful contemporary species whose biology can be used to elucidate the symbiotic evolution between commensalism and parasitism (Parmentier & Michel, 2013).

The aim of this study is to understand the life history of Carapini using three approaches: molecular phylogeny, comparative morphology and ontogeny. In the framework of our new phylogenetic study, we observed the skull development in different parasitic and commensal species to discover which morphological and/or physiological data support phylogenetic studies and how the developmental process may help in explaining the taxon history.

## Material and Methods

### Specimen collection

Specimens were obtained by snorkelling, scuba diving or trawling. The DNA retrieved from carapids (Carapidae family) represents 21 specimens, nine species and four genera, of which seven species are of the tribe Carapini. Except *Echiodon drummondi* Thompson, 1837, which was trawled at the Bay of Biscay, all specimens were hand-collected from inside their hosts by snorkelling or scuba diving in three locations: Madagascar (Toliara), French Polynesia (Moorea) and Corsica (STARESO station, Calvi). The collected specimens were brought back to the various laboratories (Aqua-Lab Laboratory of the Institut Halieutique et des Sciences Marines in Madagascar, CRIOBE laboratory in French Polynesia and STARESO Laboratory in Corsica) and placed in seawater tanks. Fish were removed from their host by dissection, or by depleting the hosts of oxygen using containers where the hosts were confined until the fish exited. Samples of integument were preserved in 100% ethanol for DNA extraction at 4 °C. Besides the collected specimens, data for the species *Carapus bermudensis* (Jones, 1874) were available from GenBank ( AP004404) and included in the present study (Table 1).

**Table 1:** GenBank accession numbers. List of species and collecting locations, along with the outgroup taxa analyzed in this study.

	Species	Ind			Genes			
			12S	16S	COI	ATPase	Cytb	18S
	CARAPINI							
	*Carapus acus*	Me1	KU681332	KU681352	KU681389	**-**	KU681395	KU681373
		Me2	KU681333	KU681353		**-**	KU681396	KU681374
	*Carapus bermudensis*	GB	AP004404	AP004404	AP004404	AP004404	AP004404	-
	*Carapus boraborensis*	FP1	-	KU681354	-	KU721887	-	KU681375
		FP2	KU681334	KU681355	-	KU721888	-	KU681376
		M1	KU681335	KU681356	-	KU721889	-	KU681377
		M2	KU681336	KU681357	-	KU721890	-	
	*Carapus homei*	FP1	KU681337	KU681358	-	KU721891	KU681397	KU681378
		FP2	KU681338	KU681359	-	KU721892	KU681398	KU681379
		M1	KU681339	KU681360	-	KU721893	KU681399	
		M2	KU681340	KU681361	-	KU721894		KU681380
	*Carapus mourlani*	FP1	KU681341	KU681362	KU681390	KU721895		KU681381
		M1	KU681342	KU681363	KU681391	KU721896	-	KU681382
		M2	KU681343	KU681364	KU681392	KU721897	-	
	*Encheliophis gracilis*	FP1	KU681345	KU681366	-	KU721898	-	KU681384
		FP2	KU681346	KU681367	-	KU721899	-	KU681385
		M1	KU681347	KU681368	-	-	-	KU681386
		M2	KU681348	KU681369	-	-	-	-
	*Encheliophis vermiops*	M1	KU681349	KU681370	KU681393	KU721900	KU681401	-
		M2	KU681350	KU681371	KU681394	KU721901	**-**	KU681387
	ECHIODONTINI							
	*Onuxodon fowleri*	FP1	KU681351	KU681372	-	KU721902	-	KU681388
	*Echiodon drummondii*	-	KU681344	KU681365	-	-	KU681400	KU681383
Outgroup	*Bassozetus zenkevitchi*	GB	AP004405	AP004405	AP004405	AP004405	AP004405	-
	*Cataetyx rubrirostris*	GB	AP004407	AP004407	AP004407	AP004407	AP004407	-
	*Diplacanthopoma brachysoma*	GB	AP004408	AP004408	AP004408	AP004408	AP004408	-

**Notes.**

Ind the number of individuals and the location of their sampling M Madagascar Me Mediterranea FP French Polynesia GB sequence obtained from GenBank “-” indicates a missing sequence

### DNA extraction, Polymerase Chain Reaction (PCR) and DNA sequencing

Genomic DNA was extracted with the commercial Invitek Spin Tissue Mini kit (Invisorb). One nuclear gene (18S rDNA) and five mitochondrial genes were analysed in the present study (Table 1). DNA fragments from the mitochondrial large ribosomal subunit (12S rDNA, ca. 770 bp; 16S rDNA, ca. 1,220 bp), ATP synthase (ATPase 8 and 6, ca. 840 nucleotides), cytochrome b (Cytb, ca. 1,005 nucleotides) and cytochrome oxidase I (COI, ca. 592 nucleotides) were amplified by PCR (see File S1). All new sequences were deposited in GenBank (Table 1).

### Outgroup, alignment, assessment of saturation, and site selection

As *Echiodon drummondi* and *Onuxodon fowleri* are considered as a sister group of Carapini in cladistic analyses based on morphological characteristics (Markle & Olney, 1990; Parmentier et al., 2000a), their monophyly and placement as a sister group of the Carapini was first tested by choosing three species of fish, *Bassozetus zenkevitchi* Rass, 1955, *Cataetyx rubrirostris* Gilbert, 1890 and *Diplacanthopoma brachysoma* Günther, 1887 as the outgroup with the 21 collected individuals as the ingroup. These fishes in the outgroup are members of Ophidiiformes and their selection was based on their phylogenetic affinities with pearlfishes (Miya et al., 2003) and the availability of the sequences of interest in databases (these fish presented five of the six sequences of interest: 18S rDNA sequences did not exist for any of these fish). As these first analyses revealed that *E. drummondi* and *O. fowleri* do form a well-defined monophyletic sister group to the Carapini, they were taken as outgroup of the Carapini in some subsequent analyses.

12S rDNA, 16S rDNA and 18S rDNA sequences were aligned using default parameters in the automatic multiple alignment program MUSCLE (Edgar, 2004); http://www.ebi.ac.uk/Tools/msa/muscle) and compared to the alignment obtained using default parameter settings in ClustalX (Thompson et al., 1997) . The two alignments differed by only 1 bp for the data set, a character that was consequently removed from the analyses. ATPase, Cytb and COI sequences were aligned according to the corresponding amino acid alignment. All alignments were inspected by eye for any obvious misalignments. The incongruence length difference (ILD) test (Farris et al., 1994) was implemented in PAUP*4.0b4a (Swofford, 2002).

The obtained matrix included 5,169 characters that were then analysed in MetaPIGA 2.1.3 (Helaers & Milinkovitch, 2010). Five sequences (two *Carapus boraborensis* (Kaup, 1856) from Madagascar and Tahiti; one *Encheliophis gracilis* (Bleeker, 1856) from Madagascar, one *Carapus homei* (Richardson, 1844) from Madagascar and one *C. mourlani* (Petit, 1834) from Madagascar) that had more than 40% of ambiguous positions were removed from the phylogenetic analyses. The two *Carapus acus* (Brunnich, 1768) from Corsica had similar sequences, and only one was retained. Invariable characters were detected in MetaPIGA and trimmed using the Gappyout trimming option (Capella-Gutiérrez, Silla-Martínez & Gabaldón, 2009). Divergence amongst sequences did not show any signs of saturation even when genes were tested separately. The resulting matrix that was subsequently analysed included 19 taxa and 5,060 characters.

### Phylogenetic analyses

The complete data set, separated genes and combinations of genes were analysed using maximum parsimony (MP), Bayesian inference (BI) and maximum likelihood (ML) methods. In all analyses, gaps were treated as missing data. MP analyses were carried out using PAUP*4.0b4a (Swofford, 2002), with all characters unordered and equally weighted. Heuristic searches were conducted using random stepwise addition of the terminals for 1,000 replicates with the tree bisection reconnection (TBR) permutation algorithm and with maximum zero-length branches collapsed. The resulting trees were summarised via strict consensus. Clade support was assessed using bootstrapping (Felsenstein, 1985) of sites on 1,000 replicates.

ML analyses were performed using the Metapopulation Genetic Algorithm (MetaGA) using the software MetaPIGA 2.1.3 (Helaers & Milinkovitch, 2010). The alignment was considered in the analyses as a whole or partitioned according to the genes (six partitions). The probability consensus pruning was used among four populations of four individuals each, and the best-fitting ML nucleotide substitution model was selected on the basis of the Akaike Information Criterion implemented in MetaPIGA. The best models that sorted from the analyses were the GTR model with rate heterogeneity without invariant for the 12S, 16S and 18S and the HKY85 for the others. As MetaPIGA (in contrast to MrBayes) does not allow applying different models to different partitions, we tested both models on the non-partitioned and partitioned alignment. To generate an estimate of the posterior probability distribution of possible trees, we performed replicated MetaGA searches and stopped automatically when a series of mean relative error values among ten consecutive consensus trees remained below 5%, with a minimum of 100 replicates (Helaers & Milinkovitch, 2010).

Bayesian analyses were performed with MrBayes v3.0b4 (Ronquist & Huelsenbeck, 2003) on the gene-partitioned alignment using the models selected on the basis of the Akaike Information Criterion implemented in MetaPIGA. We also tested the GTR +*I* +Γ on the non-partitioned data set as it was the best model proposed during a Modeltest analysis (Posada & Crandall, 1998). In addition to the analyses made on the exclusive DNA matrix, we added a seventh partition consisting of 42 morphological characteristics. Some of the morphological characteristics had already been used previously by one of us (Parmentier et al., 2000a) in a cladistic analysis retracing the phylogeny of carapids; the others are new and come from new morphological observations (see File S2). For BI analyses, four independent Markov chain Monte Carlo (MCMC) were run simultaneously for 10 × 10^6^ generations and phylogenetic trees were sampled every 100 generations. The first 25,000 trees were discarded as burn-ins. The remaining trees were used to compute Bayesian posterior probabilities (BPP) for each clade of the consensus tree. The run was repeated twice to ascertain convergence towards the same posterior parameter distribution (Huelsenbeck et al., 2002).

Alternative phylogenetic hypotheses were compared statistically by means of the Kishino–Hasegawa ML ratio test implemented in PAUP* (Kishino & Hasegawa, 1989). Also, out of the 13 existing carapin species, the molecular analyses made here only apply to eight of them. The last five species, *C. dubius* (Putnam, 1874), *C. sluiteri* (Weber, 1913), *E. chardewali* (Parmentier, 2004), *E. vermicularis* Müller, 1842 and *E. sagamianus* (Tanaka, 1908), are rare species. We thus also performed MP analyses on the matrix including the 42 morphological characteristics (File S2). The analyses were done either without constraint or with a backbone constraint where we imposed the topology sorted out from the molecular phylogenetic analyses. Clade supports were also assessed using bootstrapping (1,000 replicates).

**Figure 1:**
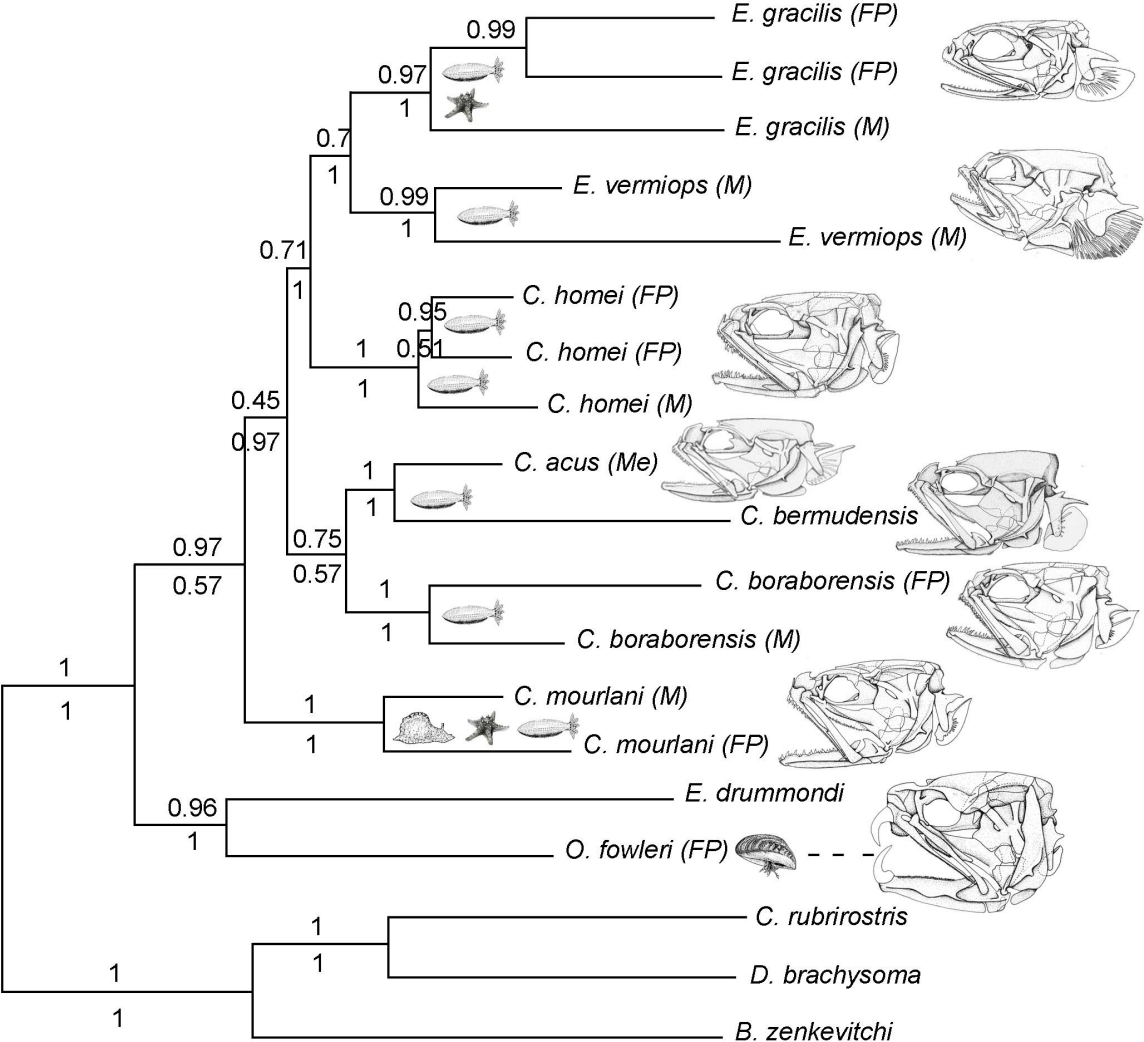
Phylogram of Carapini based on the Bayesian analysis. Phylogram showing the results of a Bayesian analysis on the full data set (six genes + morphological characteristics). The values above and below the branches indicate the posterior probabilities obtained during the Bayesian search (six genes + morphological characteristics; partition active) and ML Metaga search (six genes; unpartitioned data set), respectively. The structures of the skulls of the Carapidae are illustrated on the right. FP, French Polynesia; M, Madagascar, Me, Mediterranean.

Mesquite 3.04 (Maddison & Maddison, 2015) was used to reconstruct the evolution of characters associated with symbiosis and with the sound apparatus (swim bladder) both under maximum likelihood criteria. These characters were chosen because they provide interesting information on the phylogenetic history of the Carapini as the communication (sonic mechanism) and the habitat (host) can both have a function in prezygotic barriers (Cocroft & Ryan, 1995; Colleye et al., 2011; Grant & Grant, 1996). Likelihood methods find the ancestral state(s) that maximizes the probability of the observed states (at terminal nodes) evolving under a defined stochastic model of evolution (Pagel, 1999). The Markov k-state 1-parameter model (Lewis, 2001) was used for the ML reconstruction. The character states considered in the symbiotic status were: free living (FL), commensal of bivalves (CB), commensal of holothuroids (CH), commensal of ascidiaceans (CA), opportunistic commensal (i.e., more than two classes as hosts; OC), parasite of holothuroids (PH) and opportunistic parasite (i.e., more than two classes as hosts; OP). These character states were mapped on the backbone constrained tree that included 14 species (see Fig. 3) to which the Pyramodontinae *Snyderidia canina* was added as outgroup. The character states considered in the sound apparatus (swim bladder) were the insertion with a long tendon (LT), the insertion with a short tendon (ST) and the presence of the tendon hook system (THS). These character states were mapped on the phylogenetic tree obtained with DNA and morphological data (9 carapid species; see Fig. 1) as the character states of the other species were unknown.

## Head Development

Eight specimens of *Carapus mourlani* (TL: 7–11 cm), 13 specimens of *C. boraborensis* (TL: 13–30 cm), 15 specimens of *Carapus homei* (TL: 8–17 cm) and six specimens of *Encheliophis gracilis* (TL: 16–24 cm), all adults, were collected by scuba diving at the entrance of Opunohu Bay, Moorea, French Polynesia. The first species was found in (Asteroidea) *Culcita novaeguineae* Müller & Troshel, 1842, and the three others in three holothurian species: *Bohadschia argus* (Jaeger, 1833), *Thelenota ananas* (Jaeger, 1833) and *Thelenota anax* Clark, 1921. Fifteen *Carapus homei* (TL: 149–183 mm) tenuis and three *Carapus mourlani* (TL: 145–149 mm) tenuis were caught at night as they arrived on the reef crest of Moorea Island (Parmentier, Lo-Yat & Vandewalle, 2002b). The net used (1.5 m wide × 0.75 m in height × 5 m in length, 1 mm-wide mesh net) was similar to the one used by Dufour, Riclet & Lo-Yat, (1996). Eight *Carapus boraborensis* (TL: 88–97 mm) tenuis and three *Encheliophis gracilis* (TL: 115–118 mm) tenuis were also caught on the North coast of the Rangiroa atoll, French Polynesia (Parmentier, Lo-Yat & Vandewalle, 2002b). All specimens were stored in 70% ethanol and alizarin stained according to Taylor & VanDyke (1985).

It is difficult to obtain certain Carapidae larvae or to find larvae of the same species at different developmental states. To qualitatively compare the cranial shape between larvae and adults, drawings of the heads of the various species were adjusted proportionally to the same length assigned to the distance from the front of the mesethmoid to the back of the basioccipital. Adults and larvae are either stored in the laboratory of Functional and Evolutionary morphology of ULg or were given to different museums (Parmentier et al., 2000a; Parmentier, Castro-Aguirre & Vandewalle, 2000; Parmentier et al., 2010).

Sampling was done in public waters and did not involved any endangered and threatened marine species. Manipulations were approved by the Ethic committee of the University of Liège (agreement number 07-728).

## Results

Sequence data for the six genes totalled 5,060 bp when aligned: 757 bp in 12S rDNA, 1,192 bp in 16S rDNA, 571 bp in COI, 501 bp in ATPase, 244 bp in Cytb and 1,795 bp in 18S rDNA. Each partition was analysed separately, but the resulting trees are not shown here as they were largely congruent; the parts that were not congruent compared to the analyses of the full data set are explained below. The complete data set contained 3,513 characters that were constant, 236 variable characters but parsimony-uninformative and 1,311 parsimony-informative characters. Non-coding genes (12S rDNA, 16S rDNA and 18S rDNA) formed 74% of the aligned sequences but 57% of the parsimony-informative sites. The least informative was the nuclear 18S rDNA with only 2.2% of informative sites in its 1,795 bp alignment, while the most informative was the mitochondrial 16S rDNA with 41% of informative sites. The protein coding genes (COI, ATPase and Cytb) formed the remaining 26% of the aligned sequences but 43% of the parsimony-informative sites.

**Figure 2:**
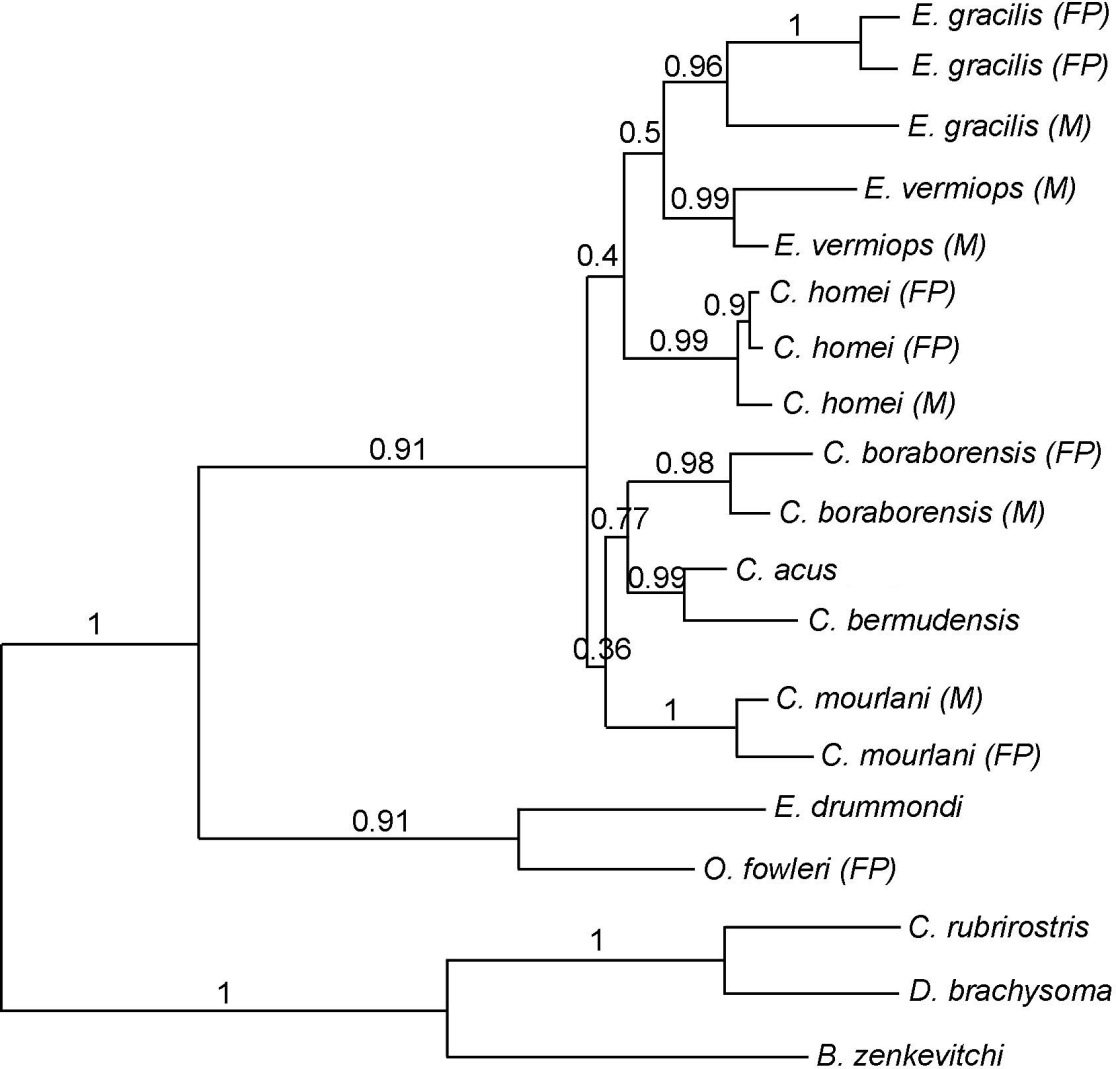
Phylogram showing the alternative hypothesis obtained in some analysis. In this phylogram, *Carapus mourlani* is the sister group of (*C. boraborensis* (*C. acus*, *C. bermudensis*)). The values above the branches indicate the posterior probabilities obtained during ML MetaGA search (six genes; partitioned data set).

Using three Ophidiiformes (*Bassozetus zenkevitchi, Cataetyx rubrirostris* and *Diplacanthopoma brachysoma*) as the out-group, all analyses (MP, MetaGA and Bayesian analyses) support the placement of a monophyletic clade made of *Onuxodon* sp. and *Echiodon* sp. at the base of the Carapidae tree with the tribe Carapini always being the sister group of it. MetaGA analyses (without partition) with the GTR + G model on the data set including the six genes, the Bayesian analysis on the six genes (with partition) and the Bayesian analysis on the data set with the seven partitions including the morphological characteristics give a unique tree as shown in Fig. 1. This tree is also retrieved when working on data sets with four genes (12S, 16S, ATPase, Cytb; ML, MP and Bayesian searches), data sets with three genes (12S, 16S, ATPase; ML, Bayesian searches) and data sets with two genes (12S, 16S; Bayesian searches). Each species was retrieved as a monophyletic cluster (Fig. 1). The genus *Carapus* forms a paraphyletic grouping in regard to the genus *Encheliophis*, which forms a monophyletic cluster (Fig. 1). The sister group of *Encheliophis* species is *C. homei*. At the base of the Carapini is *C. mourlani* and then a clade formed of three species: *C. boraborensis* being the sister group of another clade formed of *C. acus* and *C. bermudensis*. The Posterior Probabilities (PP) are >0.7 in the Bayesian search except for the support of the exclusion of *C. mourlani* from the rest of the Carapini (PP of 0.57). The low support of *C. mourlani* is due to the instability of the positioning of this clade that mostly tends to group at the base of the monophyletic group made of ((*C. acus*, *C. bermudensis*) *C. boraborensis*). An alternative hypothesis (Fig. 2) appears when working with the data set with six genes or three genes (12S, 16S and Cytb) or two genes (12S, 16S) under the MP criteria. This alternative hypothesis was also retrieved during MetaGA analysis on the partitioned data set and when excluding COI and Cytb from the analyses. As some of the genes, particularly COI and Cytb, were not strongly represented in terms of taxa, we analysed each gene separately with a MetaGA analysis. The placement of *C. mourlani* at the base of the Carapini was supported with COI, ATPase and 18S rDNA but each time with a low PP support (<70% except for COI). *Carapus mourlani* was placed at the base of the clade ((*C. acus C. bermudensis*) *C. boraborensis*) in the 16S rDNA analysis with a support of 70%. 12S rDNA analysis placed *C. mourlani* as the sister group of *C. homei* with a PP support of 39%.

In summary, these analyses support the tribe Carapini, the tribe Echiodontini and the genus *Encheliophis* as monophyletic clades, with Echiodontini being the sister group of the Carapini. They support the paraphyly of the *Carapus* genus in which ((*C. acus C. bermudensis*) *C. boraborensis*) is well supported and *C. homei* is at the base of the *Encheliophis* clade. The position of *C. mourlani* is unstable and is either observed at the base of the Carapini or at the base of the ((*C. acus C. bermudensis*) *C. boraborensis*) clade. We performed a KH test in PAUP* to compare the two alternative hypotheses (ML search with GTR = *I* + *I*). The test indicated that the best likelihood tree, the one where *C. mourlani* is at the base of the Carapini (*L* =17418.45984), is significantly different (*p* < 0.05) from the alternative hypothesis (*L* = 17418.76504) and confirms that the tree illustrated in Fig. 1 is the best.

**Figure 3:**
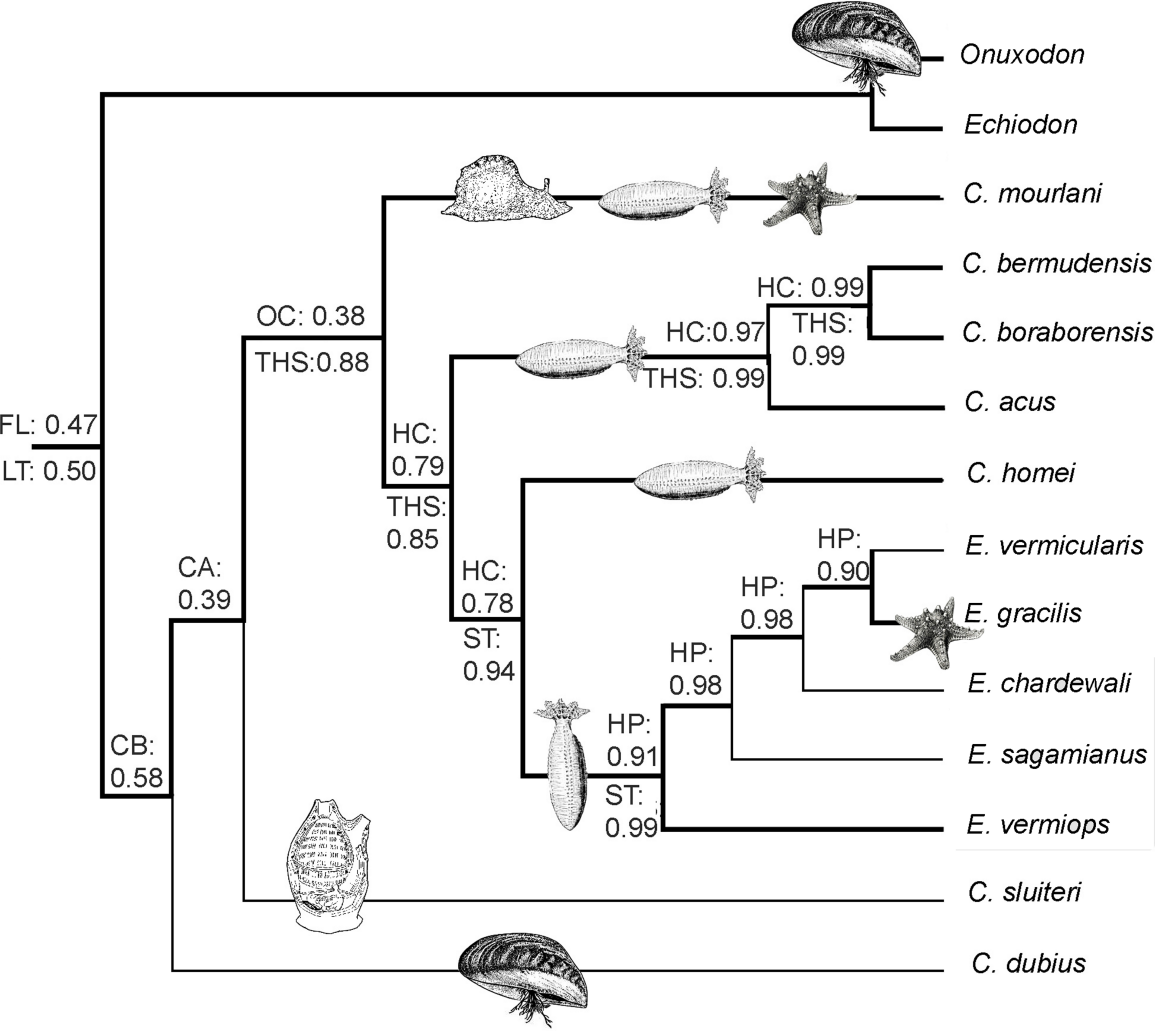
Cladorgram illustrating the results obtained during an MP search on the morphological data where a backbone constraint has been imposed on the analysis. The backbone constraint highlighted by bold branches imposes the best result obtained with molecular data (see Fig. 1). The search includes rare species for which no molecular data exist. The hosts are drawn at the bases of the clades. The best ML probabilities concerning the symbiotic status of ancestors of clades are indicated above the branches. Considered symbiotic status were: free living (FL), commensal of bivalves (CB), commensal of holothuroids (CH), commensal of ascidiaceans (CA), opportunistic commensal (i.e., more than two classes as hosts; OC), parasite of holothuroids (PH) and opportunistic parasite (i.e., more than two classes as hosts; OP). The best ML probabilities concerning the characteristics of the swim bladder of ancestors of clades are indicated below the branches. Considered characteristics of the swim bladder were: insertion with a long tendon (LT), insertion with a short tendon (ST), presence of the tendon hook system (THS).

We also performed an MP analysis of the 42 morphological characters (31 parsimony-informative) that gave the 22 best trees (MP score: 62). Five groups are in polytomy: the *Encheliophis* species that form a monophyletic grouping (BV: 98%), *Echiodon cryomargarites* (*C. acus C. homei, C. dubius*) (BV: 97%), ((*C. mourlani C. bermudensis C. sluiteri*) *C. boraborensis*) (BV: 89%) and *Onuxodon fowleri*. When the backbone constraint was imposed (see ‘Material and Methods’), the MP analysis gives a tree (Fig. 3) with 27 evolutionary steps, more than the unconstrained MP trees. The character mapping analysis gives the best ML support of 0.47 to the “free living” character state at the node at the base of the Carapinae (Fig. 3). The best supported character state at the base of the Carapini is the “Commensal of bivalve” with a ML of 0.58. Then, the character reconstruction suggests that carapids were commensals with different kinds of host, sometimes being opportunistic commensals before becoming parasites of echinoderm hosts only (Fig. 3). In parallel, the sound producing apparatus found in parasites with a short tendon insertion (Best ML value of 0.99 observed for the character state “ST” at the node at the base of the parasites) would have appeared from the tendon hook system found in commensal species only (Best ML value of 0.88 observed for the character state “THS” at the node at the base of the commensals inserted in the analysis; see ‘Material and Methods’) (Fig. 3).

**Figure 4:**
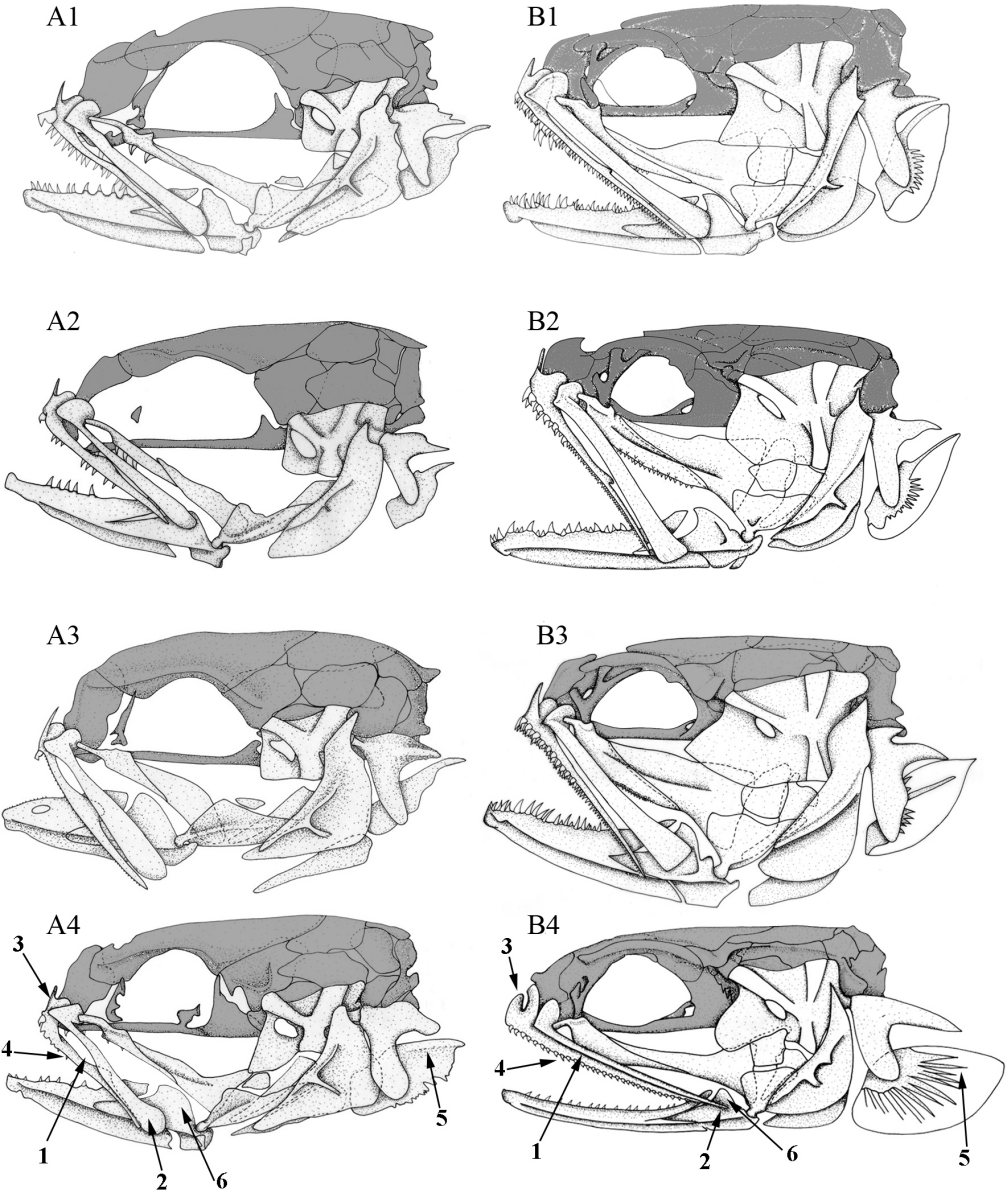
Left lateral view of different Carapini skulls. Left lateral views of the skull in different (A) larvae and (B) adult carapid species. 1, *Carapus homei*; 2, *Carapus mourlani*; 3, *Carapus boraborensis*; 4, *Encheliophis gracilis*. See results section for the numbers.

## Ontogeny

### Common developmental features of the species

In all the examined specimens, the heads of the larvae differ from those of the adults in the proportions and positions of their components (Fig. 4). These differences are more or less important depending on the species, probably because they do not have the same larval duration in open ocean (Parmentier, Lo-Yat & Vandewalle, 2002b). The neurocranium of the larvae appears proportionally higher, the eye is larger and more posterior on the skull, the opercle is smaller, the suspensorium is less extensive. The lower jaws are shorter, but more elevated, the ratio between the height of the coronoid process and the length of the lower jaw being more important than in adults. In all the studied larvae, development seems principally linked to two ontogenic trajectories.

(1) The posteroventral displacement of the quadratomandibular articulation. This transformation is accompanied by the posterior movement of the ventral part of the hyomandibula. Moreover, by the same apparent movement, the preopercle, the symplectic and the opercle become upright (Fig. 4). The posterior movement of the quadrate is also accompanied by lower jaw lengthening and later by the formation of a larger gape, as in adults.
(2) The lengthening of the otic region is more important than that of the orbital region and the hyomandibula becomes proportionally wider.

Other development patterns are as follows:

– Initially, the metapterygoid appears isolated and later joins the ventral margin of the hyomandibula. On the other hand, the mesopterygoid is initially in contact with the palatine and the quadrate only. The backwards growth of its posterior and dorsal margins then brings the mesopterygoid in contact with the metapterygoid and the hyomandibula. The latter grows ventrally and forward, while the hyomandibula thickens and the preopercular grooves broaden (Fig. 4).
– At the level of the opercle, the subopercle progressively develops an elongated dorsal spine and denticles on its posterior margin. The V-like shape of the opercle is already present in the youngest collected larvae. In the course of development, the ventral part of the V elongates.
– The number of teeth on the palatine, dentary and premaxilla increase during the development of the tenuis stage. The larvae first have poorly developed dentition. Depending on the species, teeth are missing, emerging or developing (Fig. 4). *Carapus* and *Encheliophis* larvae bear a cardiform tooth on the premaxilla (Fig. 4).

The most intriguing characteristics are found in *Encheliophis gracilis* larvae, whose morphology is closer to that of larval and adult *Carapus* than to adult *E. gracilis* (Fig. 4). Numbers following in parentheses refer to the numbered arrow in Fig. 4. In *Encheliophis* and *Carapus* larvae, (1) the maxilla and the premaxilla are free from one another, (2) the posterior part of the maxilla is broadened distally and has a rounded end, (3) the ascending process of the premaxilla is more developed than the articular process, (4) the premaxilla bears several small conical teeth, (5) the subopercle presents the outline of a dorsal spine, (6) the lower jaw is relatively robust and (7) the three gill rakers (not shown in figure) of the first ceratobranchial are well developed (Parmentier, Lo-Yat & Vandewalle, 2002b). All these features also characterise adult *Carapus* (Parmentier et al., 2000a; Parmentier et al., 1998) but are not found in *E. gracilis* adults in which (1) the maxilla and the premaxilla are attached along their entire lengths by short connective fibres, (2) the maxilla is relatively thin and tapers to a point distally, (3) the ascending process of the premaxilla is less developed than the articular process, (4) the premaxilla bears only cardiform teeth, (5) the subopercle does not display a developed dorsal spine, (6) the mandible is slender and (7) the three gill rakers of the first ceratobranchial are not developed.

### Discussion

One of the fascinating aspects of the life history of carapids lies in the understanding of their evolutionary transformation from a free living mode to a parasitic way of life. The initial relationship between carapid and host is thought to be accidental (Trott, 1970) since carapid species (e.g., *Carapus dubius*) and some species from the sister family Ophidiidae show a tendency to enter crevices and can assume tail-standing posture (Herald, 1953). The way some carapids (Schwarz et al., 2012) and *Ophidion* (Greenfield, 1968) enter their hosts and sand respectively is tail first. Among clownfishes, the first symbiotic fish was likely host specialists and subsequent speciation events led to a combination of generalist and specialist groups (Elliott et al., 1999). *Carapus dubius* is the only Carapini found in bivalves (Castro-Aguirre, Garcia-Dominguez & Balart, 1996; Parmentier, Castro-Aguirre & Vandewalle, 2000) as is the case for the *Onuxodon* species belonging to the sister taxon Echiodontini (Markle & Olney, 1990; Williams, 1984), supporting the assumption that molluscs were the first hosts of Carapidae. This species is, however, restricted to the North American coast (Markle & Olney, 1990). Another Carapini, *C. sluiteri*, was found in ascidians but it is difficult to discuss this species because to date only one specimen is known (Weber, 1913). The results from phylogenetic analysis also permit establishing a hypothesis of the relationships between Carapini and their hosts. The highest ML probabilities support other *Carapus* first opportunistic commensals (Fig. 3) as is the case for *Carapus mourlani*. This species inhabits gastropods, holothuroids and asteroids (Glynn et al., 2008). It has also the widest distribution of all Carapini, and is found from the East African to the West American coast (Markle & Olney, 1990; Parmentier, Mercier & Hamel, 2006). Carapidae were most probably first free living before having commensal relationships with hosts (bivalves and ascidians) deprived of chemical ways of defence. They were then able to enter into echinoderms and resist their chemical defences (Eeckhaut et al., 2015). Some species within this group finally developed parasitic relationships with these echinoderms.

Carapini show two morphotypes (commensal and parasite) that are closely tied to their ecological niche (Parmentier, Chardon & Vandewalle, 2002). The parasitic morphotype appeared once during the history of the fishes from the commensal morphotype. This is in accordance with the ecological niche of parasites being smaller, with a restricted number of hosts (Parmentier, Chardon & Vandewalle, 2002) and a narrower geographic distribution (Markle & Olney, 1990). Different studies have dealt with the carapini species that would be included in either *Carapus* or *Encheliophis*. Various studies have placed commensal morphotypes in *Carapus*: *C. homei*, *C. boraborensis*, *C. acus*, *C. mourlani*, *C. bermudensis*, *C. dubius* and *C. sluiteri* (Arnold, 1956; Parmentier et al., 2000a; Trott, 1970; Williams, 1984). All parasitic morphotypes have always been placed in *Encheliophis*. According to the molecular and consensus trees, *Encheliophis* is still monophyletic but *Carapus* appears to be paraphyletic. On the basis of the phylogenetic reconstruction and ontogenetic data, it is possible to draw a comprehensive scenario of the evolution of the tribe.

Adaptive radiation of a taxon results from morphological, physiological or behavioural modifications (Schluter, 2001). According to Mayr (1989), the entrance of a phyletic lineage to a new adaptive zone is linked to a morphological reorganisation leading to a new morphotype called a “rank”. Once a new rank is reached, minor morphological modifications allow the species to specialise for different ecological niches, promoting speciation (Schluter, 2009). The term “new adaptive zone” refers to a set of ecological niches that may be occupied by a group of species that exploit the same resources in a similar manner after the acquisition of morphological and/or physiological characteristics (Dumont et al., 2012; Mitter, Farrell & Wiegmann, 1988; Schluter, 2000). Morphological modifications that allow entry into a new adaptive zone can result from one or several changes of an ancestral plan (Zelditch & Fink, 1996) or from the emergence of novelties. In Carapini, the evolutionary jump from the commensal to the parasitic morphotype gave access to a new adaptive zone with which a new morphological type has appeared and radiated in different species. These morphological modifications usually appear at the end of ontogenetic development because the genetic developmental programme has no way of eliminating the reminiscent ancestral stages and is thus forced to modify them during the subsequent steps of development (Alberch et al., 1979; Mayr, 2001). The more phylogenetically closely related the species are, the later the phenotypic differentiations in development appear (Maglia, Pugener & Truer, 2001). Our data on the development of the head skeleton fulfils this assertion and concur with Haeckel’s views: new forms correspond to terminal modifications of features at the end of the ontogeny (Adriaens & Verraes, 2002; Lovtrup, 1978). The comparison between larval and adult Carapini shows that the *Encheliophis gracilis* larva is morphologically closer to the commensal morphotype than the parasitic morphotype (Fig. 4). During its ontogeny, the parasitic form goes beyond the adult stage reached by commensal form, i.e., one stage appears to have been added at the end of development (Fig. 4). These results confirm the phylogenetic results: the parasitic morphotype (=*Encheliophis* species) evolved from one of the commensal morphotypes and developed adaptations to a zone that differs from the ecological zone of the ancestral taxon. Some characteristics such as conical teeth on the upper jaw or gill rakers on the ceratobranchials 1 in the branchial basket could be the result of paedomorphosis: their development stops before the adult stage (Vandewalle et al., 1998). However, all modifications do not appear to be ontogenetic timing in *Carapus* but instead novel modifications of the shape trajectories. Modifications involving the upper jaws, lower jaws and opercle seem to be non-heterochronic modes of developmental reprogramming. More precisely, these modifications correspond to examples of “*allometric repatterning*”: ancestor and descendant differ in the trajectory of ontogenetic shape (Webster & Zelditch, 2005).

Some phylogenies reconstructed with acoustic signals were shown to be congruent with phylogenies based on morphological and molecular data (Malavasi, Collatuzzo & Torricelli, 2008). An interesting result of the present study is the position of *Carapus homei* which seems to represent a missing link between commensal and parasitic morphotypes. This result from the phylogenetic analysis (Fig. 1) is fully supported by morphological and behavioural features. *Carapus homei* shows all the characteristics associated with a commensal way of life. The morphology of the buccal and pharyngeal jaws clearly fits a diet based on elusive prey such as fish or crustaceans (Parmentier et al., 1998; Parmentier & Das, 2004; Vandewalle et al., 1998), however, the inner sound-producing apparatus is closer in structure to that of parasites. In all examined *Carapus* (*C. boraborensis*, *C. mourlani*, *C. acus*) it was recently shown that the primary sonic muscles (PSM) terminate in a complex tendon, the “tendon–hook” system (THS), which includes a “hook” that fits over a tubercle on the swim bladder (Parmentier et al., 2008). The sonic muscles contract slowly, pulling rostrally the anterior bladder. Sound is generated when the tension trips the THS and causes the bladder to snap back to its resting position (Parmentier et al., 2006b). *Carapus homei, Encheliophis gracilis* and *E. vermiops* lack the THS and, consequently have direct insertion of their PSM onto the swim bladder, meaning there are differences in the way of sounds are produced (Parmentier et al., 2008). Variation in acoustic signals could be involved in isolating mechanisms (Cocroft & Ryan, 1995; Slabbekoorn & Smith, 2002). Once again, the way the sound-producing muscles are inserted on the swim bladder supports the assumption that some characters are the result of paedomorphosis: the configuration of the short tendon is simpler than the tendon hook system.

The sound-producing system and resulting calls cannot explain the transition from the commensal to the parasitic morphotype. However, it has the advantage of providing a feature that does not seem to be under environmental constraints. Adaptive traits alone do not permit retracing the evolutionary history of a group; they only show the evolutionary stages. Orientation of evolutionary history needs the presence of characteristics apparently independent of a niche (Parmentier & Vandewalle, 2003). This seems to be the case for the sonic system in Carapini.

## Conclusion

The combination of phylogenetic, morphological and ontogenetic data indicates that parasitic species derive from commensal species. Interestingly, morphological characteristics allowing the establishment of the relationship between both ways of life are found at the level of the sound-producing mechanism, which can support the diversification of the taxa but not the acquisition of the parasite morphotype. *Carapus homei* already has the calling mechanism of the parasite, but still has a commensal way of life and the corresponding head structure. The entrance into the new adaptive zone would have been realised by at least two processes: paedomorphosis and allometric repatterning.

## Supplemental Information

10.7717/peerj.1786/supp-1 Supplemental Information 1 Detailed description of the PCR method

10.7717/peerj.1786/supp-2 Supplemental Information 2 Morphological character states and corresponding matrix. Reviewed and adapted from Parmentier, Castro-Aguirre & Vandewalle (2000b).

## Additional Information and Declarations

### DNA Deposition

The following information was supplied regarding the deposition of DNA sequences: The relevant accession numbers can be found in Table 1.
