# Supplementary material for: From commensalism to parasitism in Carapidae (Ophidiiformes): heterochronic modes of development?
Source: PeerJ. 2016 Mar 10;4:e1786. doi: 10.7717/peerj.1786 (PMC4793336; doi:10.7717/peerj.1786)
Supplement: Supplemental Information 1 [file peerj-04-1786-s001.pdf]

Supplementary material S1 - Method of amplification by PCR

DNA sequences were amplified by PCR using Ready-To-Go PCR Beads (Pharmacia). Each PCR was performed in a volume of 25µl containing 1.5 units of *Taq* DNA polymerase, 1x PCR buffer (10 mM Tris/HCl, pH 9.0), 50mM KCl, 1.5mM $MgCl_2$, 0.20mM of each dNTP, 0.6 pmol/µl of each primer, and 1µl (~10-500 ng) of genomic DNA. PCR profiles were as follows: 2 to 5 min at 95°C followed by 35 cycles of 30 sec at 95°C, 30 sec at 52-55°C (12S) or 50-53°C (16S) or 53°C (ATPase) or 46-49°C (Cytb) or 47°C (COI) or 55°C (18S), and 60 sec at 72°C, followed by a final elongation step at 72 °C for 7 min. The primers 12SbstF (5'-CAGCAGTATAAACATTA-3') and 12SbstR (5'-AGAACAGGCTCCTCTA-3') were used to amplify the first part (+/- 360 bp) of the 12S rDNA, and the primers 12SbndF (5'-ACAAACTGGGATTAGATAC-3') and 12SbndR (5'-CACCTTCCGGTACACTT-3') to amplify the second part (+/- 500 bp).
The primers L1854-16S-Cb (5'-ACACCTCGTACCTTTTGCAT-3'), modified from Inoue et al. (2000, 2001) in the aim to match the *C. bermudensis* sequence, and H2590-16S (5'-ACAAGTGATTGCGCTACCTT-3') (see Inoue et al. 2000, 2001) were used to amplify the first part (+/- 800 bp) of the 16S rDNA, and 16Sar / 16Sbr (Palumbi et al., 1997) were used to amplify the second (+/- 600 bp). The primers L8331 / H9236 (see Meyer, 1993; Quenouille et al. 2004) were used to amplify the ATPase. The primers LCO1490-Cb (5'-TCTCAACAAACCACAAAGACATTGG-3') and HCO2198-Cb (5'-TAGACCTCGGGGTGACCAAAGAATCA-3'), modified from Folmer et al. (1994) in the aim to match the *C. bermudensis* sequence, were used to amplify the COI. The primers Cytb-Cb-1F (5'-TTCGTTGTTATTCAACTACAGAAACC-3') and Cytb-Cb-1R (5'-GGGTTATTTGAGCCAGTCTCGT-3') were used to amplify the first part (+/- 600 bp) of the Cytb, whereas Cytb-Cb-2F (5'-GGCTTCTCGGTAGACAATGC-3') and Cytb-Cb-2R (5'-GAAGGGGTGTTCGACTGCTA-3') were used to amplify the second (+/- 450 bp). Except H2590-16S, 16Sar / 16Sbr and L8331 / H9236, primers were newly designed for the present study (D. Lanterbecq). The 18S rDNA was amplified in three overlapping fragments of about 600 nucleotides each using primers from Eeckhaut et al. (2000). Amplification products were purified either with the Qiaquick PCR kit (QIAGEN) and Invisorb or from 1% agarose gels (Quantum Prep Freeze 'N Squeeze, Biorad). Both strands of each PCR product were directly sequenced using the BigDye™ Terminator Cycle Sequencing Kit (Applied Biosystems) and products were separated electrophoretically

using an Applied Biosystems 3100 automated sequencer. Sequences of the rDNA and 16S rDNA gene fragments were successfully obtained for 21 individuals. Sequences of COI, ATPase, Cytb and 18S rDNA were successfully obtained for 7, 16, 12, 16 individuals, respectively (see Table 2). Sequences were edited with Codon CodeAligner (Codon Code Corporation, Dedham, MA) and Se-Al v2.0a11 (Rambaut, 1996).
