# Supplementary material for: From commensalism to parasitism in Carapidae (Ophidiiformes): heterochronic modes of development?
Source: PeerJ. 2016 Mar 10;4:e1786. doi: 10.7717/peerj.1786 (PMC4793336; doi:10.7717/peerj.1786)
Supplement: Supplemental Information 2 — Reviewed and adapted from Parmentier, Castro-Aguirre & Vandewalle (2000b). [file peerj-04-1786-s002.pdf]

Supplementary file S2

List and state of the different morphological characters. See Parmentier et al. (2002, 2010) for detailed information.

number	character	State
**1**	Upper jaws not hidden by skin	0
	Upper jaws hidden by skin	1
**2**	ascending processes of the premaxilla missing	0
	Not the case	1
**3**	ascending and lateral processes of the premaxilla clearly distinct and V-shaped	0
	ascending and lateral processes of the premaxilla parallel	1
**4**	No foramen on the premaxilla	0
	Foramen on the premaxilla	1
**5**	distal tip of the maxillary large and rounded	0
	distal tip of the maxillary pointed	1
**6**	Ligament between maxilla and articulo-angular	0
	Ligament absent	1
**7**	No Connective fibers between maxilla and premaxilla	0
	Connective fibers between maxilla and premaxilla	1
**8**	Coronoid process = 25 to 30% of the mandible length	0
	Coronoid process = 16 to 18% of the mandible length	1
**9**	Lateral ethmoid with lateral branch (= U-shaped)	0
	Lateral ethmoid without lateral branch	1
**10**	Suboperculum having a long dorsocaudal digitation	0
	Suboperculum with numerous small caudal digitations	1
**11**	Pharyngobranchial 2 larger than pharyngobranchial 4	0
	Pharyngobranchial 2 smaller than or equal in size to pharyngrobranchial 4	1
**12**	Cartilaginous interarcual element	0
	Bony interarcual element	1
**13**	Site of articulation between interarcual element and epibranchial 1 high or midway on the epibranchial 1	0
	Site of articulation between interarcual element and epibranchial 1 very low on the epibranchial 1	1
**14**	Three well developed gill rakers on ceratobranchial 1	0
	Three short gill rakers on ceratobranchial 1	1
**15***	Pleural ribs	0
	No pleural ribs	1
**16**	Swimbladder plate on third centrum not greatly expanded posteriorly	0
	Swimbladder plate on third centrum greatly expanded posteriorly, forming a uniform convex plate	1
**17**	External row of straight, conical teeth on premaxilla	0
	No external row of straight, conical teeth on premaxilla	1
**18**	No cardiform teeth on premaxilla	0
	Cardiform teeth on premaxilla	1
**19**	Small conical teeth on the posterior half of the premaxilla	0
	Lack of small conical teeth on the posterior half of the premaxilla	1
**20**	Conical teeth on the anterior half of the premaxilla	0
	No conical teeth on the anterior half of the premaxilla	1
**21**	No enlarged conical teeth at anterior end of the premaxilla	0
	Enlarged conical teeth at the anterior end of the premaxilla	1
**22**	Caniniform teeth (fangs) at distal tips of jaws	1
	No caniniform teeth (fangs) at distal tips of jaws	0
**23**	No diastema on upper and lower jaws	0
	Diastema on upper and lower jaws	1

**24**	No small teeth externally bordered by larger, inward-curving teeth on the dentary	0
	Small teeth externally bordered by larger, inward-curving teeth on the dentary	1
**25**	No uniserial row of big conical teeth on the dentary	0
	Uniserial row of big conical teeth on the dentary	1
**26**	No uniserial row of small conical teeth on the dentary	0
	Uniserial row of small conical teeth on the dentary	1
**27**	No prominent tooth on vomer	0
	Prominent tooth on vomer	1
**28**	Teeth on basibranchial 3	0
	No teeth on basibranchial 3	1
**29**	No teeth on hypobranchial 3	0
	Teeth on hypobranchial 3	1
**30**	No cardiform teeth on lower pharyngeal jaws	0
	Cardiform teeth on lower pharyngeal jaws	1
**31**	Insertion of A1β on rostro-medial side of the maxillary	0
	Insertion of A1β on rostro-dorsal side of the maxillary	1
**32**	Insertion of the adductor branchialis 5 on epibranchial 4	0
	Insertion of the adductor branchialis 5 on epibranchials 3 and 4	1
**33**	Lack of four recti dorsales muscles	0
	Presence of four recti dorsales muscles	1
**34**	Primary sonic mluscles present	0
	No primary sonic muscles	1
**35**	No central constriction at level of swim bladder	0
	Swim bladder central constriction	1
**36**	Rocker bone absent	0
	Rocker bone present	1
**37**	Pectoral fins present	0
	Pectoral fins absent	1
**38**	Scapula present	0
	Scapula absent	1
**39**	Lateral ethmoid with three shelves	0
	Lateral ethmoid with two shelves	1
**40**	Palatine teeth straight	0
	Palatine teeth curved anteriorly	1
**41**	No parallel tunic ridges on the swimbladder	0
	Parallel tunic ridges on the swimbladder	1
**42**	Swimbladder without “tendon-hook” system	1
	Swimbladder with a “tendon-hook” system	0

*Pleural ribs is used in the sense given by Markle 1989.

Supplementary file S2

Matrix of the different characters. Data of *Carapus sluiteri* are not confirmed; their feature states do not depend on this work. C.: *Carapus*, E.: *Encheliophis*; Ec.: *Echiodon* O.: *Onuxodon*; S.: *Snyderidia*

Charactère	1	2	3	4	5	6	7	8	9	10	11	12	13	14	15	16	17	18	19	20	21	22	23	24	25	26	27	28	29	30	31	32	33	34	35	36	37	38	39	40	41	42
S. canina	0	0	0	0	0	0	0	0	0	0	0	0	0	0	0	0	1	0	0	0	0	1	0	0	1	0	1	0	0	0	0	0	0	0	0	0	0	0	0	0	0	0
O. fowleri	0	1	0	1	0	0	0	0	0	0	0	1	0	0	1	1	0	0	0	0	0	1	1	0	0	0	0	1	0	0	0	0	0	0	0	1	0	0	0	0	0	0
C. mourlani	0	0	0	0	0	0	0	0	1	0	0	1	1	0	1	1	0	1	0	0	1	0	0	1	0	0	0	1	1	0	0	0	1	0	1	0	0	0	0	0	0	1
C. bermudensis	0	0	0	0	0	0	0	1	1	0	0	1	1	0	1	1	0	1	0	0	1	0	0	1	0	0	0	1	1	0	0	0	1	0	1	0	0	0	0	0	0	?
C. acus	0	0	0	0	0	0	0	0	1	0	0	1	1	0	1	1	0	1	0	0	1	0	0	1	0	0	0	1	1	0	0	0	1	0	1	0	0	0	0	0	0	1
C. sluiteri	0	?	?	0	0	0	?	?	1	?	?	1	1	0	1	1	0	1	0	0	1	0	0	1	0	0	0	?	?	?	?	?	?	?	1	0	?	?	?	?	0	?
C. homei	0	0	0	0	0	0	0	0	1	0	0	1	1	0	1	1	0	1	0	0	1	0	0	1	0	0	0	1	0	0	0	0	1	0	0	0	0	0	0	0	0	0
C. boraborensis	0	0	0	0	0	0	0	0	1	0	0	1	1	0	1	1	0	1	0	0	1	0	0	1	0	0	0	1	1	0	0	0	1	0	0	0	0	0	0	0	0	1
C. dubius	0	0	0	0	0	0	0	0	1	0	0	1	1	0	1	1	0	1	0	0	1	0	0	1	0	0	0	1	1	0	1	0	1	0	0	0	0	0	0	0	0	?
E. vermicularis	1	0	1	0	1	1	1	0	1	1	1	1	1	1	1	1	0	1	1	0	0	0	0	0	0	1	0	0	0	0	1	1	1	0	0	0	1	0	1	0	0	?
E. gracilis	1	0	1	0	1	1	1	1	1	1	1	1	1	1	1	1	0	1	1	1	0	0	0	0	0	1	0	0	0	1	1	1	1	0	0	0	0	0	1	0	0	0
E. sagamianus	1	0	0	0	1	1	1	1	1	1	0	1	1	1	1	1	0	1	1	0	0	0	0	0	0	1	0	1	1	0	1	1	1	0	0	0	0	0	0	0	0	?
E. vermiops	1	0	0	0	0	1	1	0	1	1	0	1	1	0	1	1	0	1	0	0	0	0	0	0	0	1	0	1	0	0	?	?	?	0	0	0	0	0	1	1	0	0
E. chardewalli	1	0	1	0	1	1	1	1	1	1	?	1	1	1	1	1	0	1	1	0	0	0	0	0	0	1	0	0	0	?	?	?	?	0	0	0	1	1	0	0	0	?
Ec. cryomargarites	0	0	0	0	0	0	0	?	?	?	?	1	0	0	?	?	0	0	0	0	0	1	1	0	0	0	0	1	0	0	0	?	?	0	0	0	0	0	0	0	1	0
